# Differential Associations Between Two Markers of Probable Sarcopenia and Continuous Orthostatic Hemodynamics in The Irish Longitudinal Study on Ageing

**DOI:** 10.1093/gerona/glac243

**Published:** 2022-12-08

**Authors:** Eoin Duggan, Caoileann H Murphy, Silvin P Knight, James R C Davis, Aisling M O’Halloran, Rose Anne Kenny, Roman Romero-Ortuno

**Affiliations:** The Irish Longitudinal Study on Ageing (TILDA), School of Medicine, Trinity College Dublin, Dublin, Ireland; Discipline of Medical Gerontology, School of Medicine, Trinity College Dublin, Dublin, Ireland; The Irish Longitudinal Study on Ageing (TILDA), School of Medicine, Trinity College Dublin, Dublin, Ireland; Teagasc, Food Research Centre, Ashtown, Dublin, Ireland; The Irish Longitudinal Study on Ageing (TILDA), School of Medicine, Trinity College Dublin, Dublin, Ireland; Discipline of Medical Gerontology, School of Medicine, Trinity College Dublin, Dublin, Ireland; The Irish Longitudinal Study on Ageing (TILDA), School of Medicine, Trinity College Dublin, Dublin, Ireland; Discipline of Medical Gerontology, School of Medicine, Trinity College Dublin, Dublin, Ireland; The Irish Longitudinal Study on Ageing (TILDA), School of Medicine, Trinity College Dublin, Dublin, Ireland; Discipline of Medical Gerontology, School of Medicine, Trinity College Dublin, Dublin, Ireland; The Irish Longitudinal Study on Ageing (TILDA), School of Medicine, Trinity College Dublin, Dublin, Ireland; Discipline of Medical Gerontology, School of Medicine, Trinity College Dublin, Dublin, Ireland; The Irish Longitudinal Study on Ageing (TILDA), School of Medicine, Trinity College Dublin, Dublin, Ireland; Discipline of Medical Gerontology, School of Medicine, Trinity College Dublin, Dublin, Ireland

**Keywords:** 5-Chair stands test, Grip strength, Orthostatic hypotension, Sarcopenia

## Abstract

**Background:**

Sarcopenia and orthostatic hypotension are growing age-related health burdens associated with adverse outcomes, including falls. Despite a possible pathophysiological link, the association between the 2 disorders is not well elucidated. We sought to investigate this relationship in The Irish Longitudinal Study on Ageing (TILDA).

**Methods:**

Data from 2 858 participants at wave 3 of TILDA were analyzed. Probable sarcopenia was defined as per the European Working Group on Sarcopenia in Older People revised definition cutoffs (hand grip strength [HGS] <27 kg in men, <16 kg in women, and/or 5-chair stand test [5CST] time >15 seconds). Participants underwent an active stand orthostatic test with continuous blood pressure (BP) monitoring. Multilevel mixed-effects models, controlling for possible confounders, were used to assess the effect of probable sarcopenia by HGS and 5CST criteria on the change in BP after standing.

**Results:**

HGS- and 5CST-defined probable sarcopenia were independently associated with an attenuated BP recovery at 10–20 seconds poststand (systolic BP: β −0.54, *p* < .001; β −0.25, *p* < .001). On average, those meeting HGS probable sarcopenia criteria had a significantly lower BP at 20, 30, and 40 seconds (differences in systolic BP: −5.01 mmHg, −3.68 mmHg, −2.32 mmHg, *p* < .05 for all). Those meeting 5CST probable sarcopenia criteria had a significant difference in systolic BP at 20 seconds (−1.94 mmHg, *p* = .002) but not at 30 or 40 seconds.

**Conclusion:**

Probable sarcopenia had a significant association with delayed orthostatic BP recovery, with HGS-defined probable sarcopenia having a stronger association than 5CST-defined probable sarcopenia. Results support a modest but significant pathophysiological link between probable sarcopenia and orthostatic hypotension.

Sarcopenia, a condition characterized by accelerated loss of muscle mass and function, is increasingly recognized as a cause of falls, adverse health outcomes, and mortality (1). Some have labeled it the biological substrate of physical frailty and propose that it is the pathway through which the consequences of physical frailty develop (2,3).

Orthostatic hypotension (OH), classically defined as a sustained drop of 20 mmHg systolic blood pressure (SBP) and/or 10 mmHg diastolic blood pressure (DBP) within 3 minutes of standing (4), is a common condition in older people, is increasing in prevalence (5) and is a well-recognized cause of falls (6), with risk of resultant hospitalization and institutionalization (7).

The association between OH and frailty has been well established (8,9). However, the relationship between sarcopenia and OH is less well characterized (10). This is somewhat surprising as one might hypothesize a direct link between the 2, namely via the skeletal muscle pump, which aids venous return from the lower limbs via rhythmic muscle activity during standing (11). The reduced muscle mass of sarcopenia might be expected to attenuate the effects of the skeletal muscle pump, potentially increasing the risk of OH.

One previous study found that severe sarcopenia, as classified by the European Working Group on Sarcopenia in Older People (EWGSOP) revised definition (12), was a significant predictor of OH at 1 minute and systolic OH at 5 minutes, when compared to the robust, probable sarcopenia and sarcopenia groups (13). They, however used the head-up tilt test, which gives a different hemodynamic pattern to the active stand (14) and may reduce the contribution of the skeletal muscle pump. Another smaller study found that EWGSOP-defined sarcopenia, was an independent predictor of classical OH (15). They utilized the active stand test but adjusted for only a small number of confounders. Both studies used oscillometric blood pressure (BP) and so were unable to study the early phase of BP recovery (ie, the first minute post active stand).

With the limitations of these previous studies, we sought to further investigate the relationship between sarcopenia and OH using a large, well-characterized sample with continuous noninvasive beat-to-beat BP measurements from The Irish Longitudinal Study on Ageing (TILDA).

## Method

### Study Population

TILDA is an ongoing, nationally representative longitudinal cohort study of adults aged 50 years and older (16,17). This study utilized baseline data from wave 3 of TILDA (March 2014–December 2015), with follow up of participants at wave 5 (2018). For wave 3, participants underwent a computer aided personal interview (CAPI) with a trained interviewer, which addressed questions on socioeconomic, physical, cognitive, and mental health factors. Participants were also invited to attend a dedicated health center for a research nurse-led comprehensive health assessment. At wave 5 only the CAPI was undertaken. Ethical approval for each wave was provided by the Faculty of Health Sciences Research Ethics Committee at Trinity College Dublin. All participants provided written informed consent, and the study adhered to the Declaration of Helsinki. Participants with complete data for the active stand test, probable sarcopenia measurements and covariates, and without a self-reported diagnosis of idiopathic Parkinson’s disease were included in the analysis.

### Assessment of Probable Sarcopenia

Probable sarcopenia was defined as per the EWGSOP revised definition (12) with cutoffs for hand grip strength (HGS) of less than 27 kg in men and 16 kg in women and/or time taken on the 5-chair stand test (5CST) of greater than 15 seconds. HGS was measured with a Baseline® Hydraulic Hand Dynamometer (Fabrication Enterprises Inc., White Plains, NY) while standing. The maximum value of 2 attempts on each hand was taken. For the 5CST, the time was measured to the nearest centi-second for a participant to stand up from a sitting position and sit back down again 5 times from a standard chair (approximate seat height of 46 cm), as fast as possible, without the use of arms.

### Assessment of Orthostatic Hypotension

Participants underwent an active stand orthostatic test with noninvasive beat-to-beat BP monitoring using digital photoplethysmography (Finometer® MIDI device, Finapres Medical Systems BV, Amsterdam, The Netherlands). Participants rested supine for 10 minutes in a quiet room at ambient temperature before being asked to stand quickly, unaided. They remained standing quietly while BP and heart rate continued to be measured for 3 minutes. The beat-to-beat BP data were preprocessed to remove artifacts, perform 10-second moving average filtering and feature extraction as described previously (18). Baseline supine SBP and DBP values were derived from the average of values occurring 30–60 seconds prior to standing. OH at 30 seconds (OH30), an important marker of impaired BP recovery (19,20) was defined as a drop of SBP ≥ 20 mmHg and/or DBP ≥ 10 mmHg from baseline values. Consensus OH (cOH) was defined as a sustained (10 seconds) drop in SBP of ≥ 20 mmHg and/or DBP ≥ 10 mmHg occurring between 60 and 180 seconds. If there was baseline hypertension (≥140/90 mmHg) then a drop of ≥30/15 mmHg was necessary (4).

### Covariates

Potential covariates were explored with a directed acyclic graph, and variables that may confound the relationship between sarcopenia and orthostatic BP were identified and included as covariates in the model. These included: age; sex; height; weight; the presence or absence of self-reported hypertension, diabetes, and heart failure; self-reported cardiovascular medications: World Health Organization Anatomical Therapeutic Chemical (ATC) Classification codes C01—antiarrhythmics, C02—antihypertensives, C03—diuretics, C04—vasodilators, C07—beta-blocking agents, C08—calcium-channel blockers, C09—agents acting on renin–angiotensin system; self-reported psychotropic medications: ATC codes N03A—antiepileptics, N05—antipsychotics, anxiolytics, hypnotics and sedatives, N06A antidepressants; and presence or absence of fear of falling with activity limitation. Variables that mediated the relationship between sarcopenia and OH but lay on the causal pathway were not included as covariates in the model.

### Longitudinal Follow-Up

To examine the impact of probable sarcopenia defined by both 5CST and HGS, longitudinal outcomes were assessed at wave 5. The outcomes included were: self-reported recurrent falls (2 or more falls in the last year), fear of falling with activity limitation, self-reported hospital admission (1 or more hospital admissions in the last year), and mortality (from linkage with official death registration data (21)).

### Statistical Analysis

Statistical analysis was performed in Stata version 15.1 (Stata Corp LLC, College Station, TX). Descriptive statistics were presented as the mean and standard deviation of continuous variables and count and percentage of categorical variables. Differences in means and frequencies between the non-sarcopenia and probable sarcopenia groups were evaluated with the *t* test and chi-squared test, respectively.

To assess the effect of probable sarcopenia on the change in SBP and DBP from baseline after standing, multilevel mixed-effects models were used, with fixed and random effects accounting for the repeated measurements within participants. Piecewise linear splines modeling time points 0–10, 10–20, 20–30, 30–40, and 40–180 seconds after standing were used as previously described (22,23). Residual variance was modeled with a first order autoregressive process to account for the strong correlation between adjacent timepoints. The spline parameters were included as independent parameters in the mixed-effects model along with the interaction term of probable sarcopenia, 5CST criteria or HGS criteria (0—no, 1—yes), at each time segment. The potential confounders listed previously were entered as covariates in the model. Where there was a significant change in terms of the interaction of the probable sarcopenia parameters on the BP response over specific time intervals, contrast analysis was used to assess differences in the mean change from baseline in BP at the relevant time points. Marginal effect plots (holding covariates at their means) were produced for the predicted mean change from baseline in SBP and DBP for those with and without probable sarcopenia by 5CST and HGS criteria.

Multivariable logistic regression models were used to investigate the prediction of OH30 by probable sarcopenia by 5CST and HGS criteria, while controlling for confounders. Finally, differences in follow-up outcomes for the HGS- and 5CST-defined probable sarcopenia groups compared with the non-sarcopenic group were examined with the chi-squared test.

## Results

**Figure 1.**
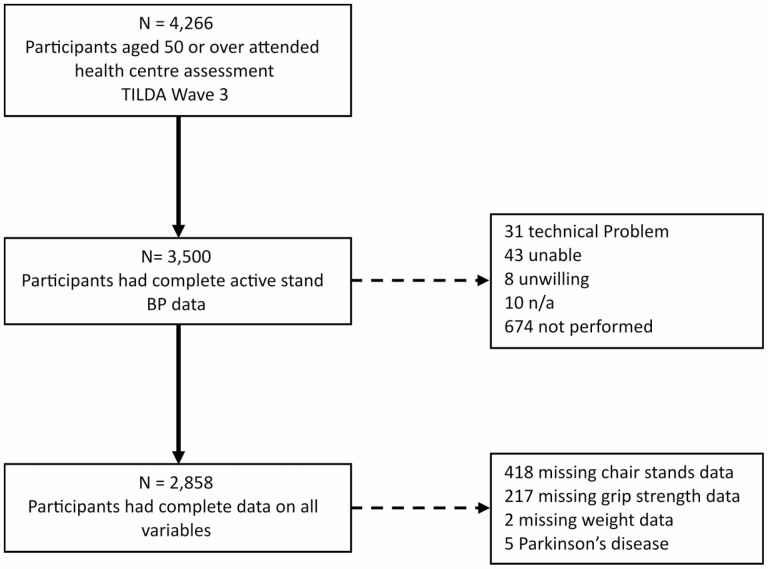
Flowchart of participants. BP = blood pressure; TILDA = The Irish Longitudinal Study on Ageing.

Complete data were available for 2 858 participants (Figure 1). The demographics and characteristics of the sample, grouped by probable sarcopenia criteria are detailed in Table 1. Compared to the non-sarcopenic group, those meeting the HGS criteria for probable sarcopenia were older, with a higher proportion of women, shorter stature, lower weight, a higher prevalence of diabetes, were more likely to be on cardiovascular and psychotropic medications and had a higher prevalence of cOH.

**Table 1.** Characteristics of the Study Population Grouped by Probable Sarcopenia Cutoffs Versus Non-Sarcopenic: 5-Chair Stands Test (5CST) Time > 15 Seconds; Hand Grip Strength (HGS) < 16 kg (Women)/27 kg (Men)

Demographics and Characteristics	5CST Criteria No		5CST Criteria Yes		HGS Criteria No		HGS Criteria Yes	
*N*	2 135	74.7%	723	25.3%	2 671	93.5%	187	6.5%
Age (years; mean/*SD*)	63.1	7.3	66.5	8.1 ***	63.5	7.3	70.2	9.0 ***
Female (*n*/%)	1 138	53.3%	387	53.5%	1 443	54.0%	82	43.9% **
Tertiary education (*n*/%)	962	45.1%	24	40.7%	1 194	44.7%	62	33.2% ***
Height (cm; mean/*SD*)	166.2	9.1	166.4	9.3	166.4	9.1	163.7	9.2 ***
Weight (kg; mean/*SD*)	77.7	14.9	79.1	15.4 *	78.2	15.1	75.9	15.0 *
Hypertension (*n*/%)	626	29.3%	249	34.4% *	808	30.3%	67	35.8%
Diabetes (*n*/%)	112	5.3%	59	8.2% **	153	5.7%	18	9.6% *
Heart failure (*n*/%)	9	0.4%	5	0.7%	13	0.5%	1	0.5%
Cardiovascular medications (*n*/%)	737	34.5%	308	42.6% ***	955	35.8%	90	48.1% **
Psychotropic medications (*n*/%)	231	10.8%	105	14.5% **	302	11.3%	34	18.2% **
Fear of falling with activity limitation (*n*/%)	86	4.0%	60	8.3% ***	129	4.8%	17	9.1% *
Consensus OH (*n*/%)	154	7.2%	58	8%	191	7.2%	21	11.2% *

*Notes*: *T* test/Chi-squared test of probable sarcopenia versus non-sarcopenia for each criterion. OH = orthostatic hypotension; *SD* = standard deviation.

*p < .05; **p < .01; ***p < .001.

**Figure 2.**
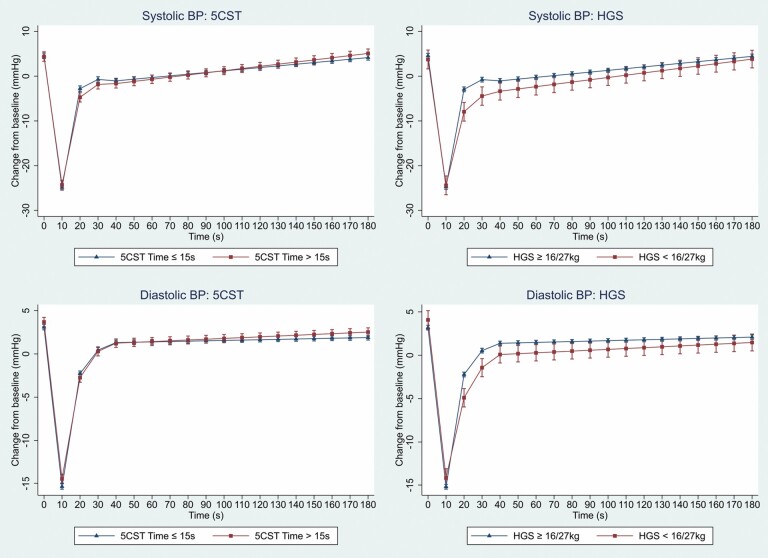
Predicted means and 95% confidence intervals, from mixed effect models, for systolic and diastolic blood pressure change (mmHg) after standing, stratified by probable sarcopenia status for both 5-chair stands test (5CST) and hand grip strength (HGS) criteria (5CST > 15 seconds, HGS < 16 kg women/27 kg men). Model adjusted for age, sex, height, weight, hypertension, diabetes, heart failure, cardiovascular and psychotropic medications, and fear of falling with activity limitation. BP = blood pressure.

Figure 2 shows the mean change in SBP and DBP from baseline, predicted from the mixed-effects models, stratified by those meeting 5CST and HGS probable sarcopenia criteria. Both 5CST- and HGS-defined probable sarcopenia were independently associated with an attenuated SBP and DBP recovery in the 10–20 second period. This agrees with the model output (Table 2), with the negative coefficient indicating a decrease in the rate of recovery. Notably, the effect size of HGS was twice that of 5CST. Those meeting the HGS criteria had a significantly lower SBP and DBP at 20, 30, and 40 seconds compared to those who did not: differences in SBP: −5.01 mmHg, −3.68 mmHg, −2.32 mmHg, *p* < .05 for all; differences in DBP: −2.68 mmHg, −1.96 mmHg, −1.30 mmHg, *p* < .05 for all. Those meeting the 5CST criteria had a significant difference in SBP at 20 seconds (−1.94 mmHg, *p* = .002) but not at 30 or 40 seconds.

**Table 2.** Coefficients and 95% Confidence Intervals for Probable Sarcopenia Criteria (5-Chair Stands Test [5CST] Time > 15 Seconds, Hand Grip Strength [HGS] < 16 kg Women/27 kg Men) From Mixed-Effects Models for Change in Systolic and Diastolic Blood Pressure (mmHg) From Baseline After Standing at Time Intervals 0–10 Seconds, 10–20 Seconds, 20–30 Seconds, 30–40 Seconds, and 40–180 Seconds

	0–10 Seconds β (95% CI)	10–20 Seconds β (95% CI)	20–30 Seconds β (95% CI)	30–40 Seconds β (95% CI)	40–180 Seconds β (95% CI)
Systolic BP (mmHg)					
5CST-defined probable sarcopenia	0.07 (<0.01, 0.14)*	−0.25 (−0.31, −0.18)***	0.08 (0.02, 0.15)*	0.05 (−0.02, 0.11)	0.01 (<0.01, 0.02)**
HGS-defined probable sarcopenia	0.12 (<0.01, 0.23)	−0.54 (−0.65, −0.42)***	0.13 (0.02, 0.25)*	0.14 (0.02, 0.25)*	0.01 (<0.01, 0.02)
Diastolic BP (mmHg)					
5CST-defined probable sarcopenia	0.03 (<0.01, 0.07)	−0.14 (−0.18, −0.10)***	0.03 (−0.01, 0.07)	0.01 (−0.03, 0.04)	<0.01 (<0.01, 0.01)**
HGS-defined probable sarcopenia	0.02 (−0.05, 0.08)	−0.37 (−0.44, −0.31)***	0.07 (0.01, 0.14)*	0.07 (<0.01, 0.13)*	<0.01 (<0.01, 0.01)

*Notes*: Model adjusted for age, sex, height, weight, hypertension, diabetes, heart failure, cardiovascular and psychotropic medications, and fear of falling with activity limitation. BP = blood pressure; CI = confidence interval.

*p < .05; **p < .01; ***p < .001.

In the initial phase (0–10 seconds), probable sarcopenia by 5CST criteria had a weak effect on SBP but not DBP drop, while in the steady state (40–180 seconds), both 5CST and HGS had very little effect on SBP and DBP (Table 2).

HGS-defined probable sarcopenia was an independent predictor of OH30 in a multivariable logistic regression model (Table 3), whereas 5CST was not (odds ratio 0.93, *p* = .538).

**Table 3.** Multivariable Logistic Regression for Orthostatic Hypotension at 30 Seconds (OH30), for Probable Sarcopenia Defined by Hand Grip Strength (HGS) Criteria (<16 kg Women/27 kg Men)

OH30	Odds Ratio	*p*	95% Confidence Interval	
HGS-defined probable sarcopenia	1.64	.012	1.11	2.42
Age	1.05	.000	1.03	1.06
Female	1.23	.247	0.87	1.74
Height	1.03	.003	1.01	1.05
Weight	0.98	.001	0.97	0.99
Hypertension	1.34	.092	0.95	1.88
Diabetes	1.49	.063	0.98	2.27
Heart failure	1.88	.304	0.56	6.29
Cardiovascular medications	1.22	.272	0.86	1.72
Psychotropic medications	1.28	.140	0.92	1.78
Fear of falling with activity limitation	0.63	.106	0.36	1.10

At follow-up in wave 5, those with probable sarcopenia by the HGS criteria in wave 3 had higher proportions of recurrent falls (10.5% vs 5.7%, *p* = .014), fear of falling with activity limitation (12.4% vs 6.9%, *p* = .011), hospital admission (24.2% vs 12.5%, *p* < .001), and death (4.8% vs 1.9%, *p* = .006) when compared to the non-sarcopenic group. Those with probable sarcopenia by 5CST criteria had a higher proportion of recurrent falls (8.0% vs 5.3%, *p* = .012), fear of falling with activity limitation (11.1% vs 5.9%, *p* < .001) but not hospital admission (15.3% vs 12.5%, *p* = .07) or death (2.8% vs 1.8%, *p* = .126), when compared with the non-sarcopenic group.

## Discussion

In this study, probable sarcopenia was associated with a significantly attenuated BP recovery from nadir after standing. The effect of HGS-defined probable sarcopenia was twice as strong as that of 5CST-defined probable sarcopenia. Those with HGS-defined probable sarcopenia had significantly lower SBP and DBP at 20, 30, and 40 seconds after standing, and HGS-defined probable sarcopenia was an independent predictor of OH30. The follow-up results at wave 5 show that 4 years later, there was a higher occurrence of falls, hospital admissions and mortality in the group categorized as probable sarcopenia by HGS criteria. This is the first study to examine the association between probable sarcopenia and orthostatic hemodynamics measured with continuous beat-to-beat digital photoplethysmography allowing precise characterization of BP phenotype. The results are further strengthened by a large and well-characterized sample allowing adjustment for potential confounders, and longitudinal follow-up 4 years after the initial assessment.

These results point to possible subtle differences in the underlying pathophysiology of the effects of probable sarcopenia on BP recovery after standing and are interesting in that the upper limb measures of probable sarcopenia seemed to predict orthostatic hemodynamics better than lower limb measures. This is somewhat surprising as a priori one might expect the lower limb to have a greater effect on BP, via the skeletal muscle pump, than the upper limb. There are 2 main possibilities for this result. The upper limbs may be more important in terms of the skeletal muscle pump than the lower limbs, or 5CST time may not be as good a measure of lower limb muscle strength. Indeed, others have raised the fact that 5CST tests not only strength but balance, proprioception, mobility, and endurance (24). To the best of our knowledge, there is no literature examining the relative contributions of the upper and lower limbs to the skeletal muscle pump in orthostatic BP recovery. Given that HGS correlates better with muscle mass than 5CST time (25,26), it may be a better proxy for lower limb strength and correlate better with orthostatic BP recovery.

Probable sarcopenia, as defined by 5CST criteria, had a weak but positive association with SBP in the initial 10 second period. However, as SBP was dropping at this time, this positive association means that those meeting the 5CST criteria had a less steep fall in their SBP after standing. This differs from the findings from Mol et al. (27), who found that the rate of SBP decline but not the magnitude was associated with 5CST time in seconds. However, there were significant differences in methodology: Mol et al. used 5-second averaging of BP and examined the first 15 second period, whereas we used 10-second averaging and looked at the first 10 second period, which may account for the differences. Furthermore, the mechanisms behind the initial drop in BP are not fully elucidated (28) and recent evidence has found better outcomes in those with initial OH (29,30), suggesting that a larger initial drop is physiological rather than pathological.

What is clearer, however, is the importance of BP recovery from nadir (31,32). The strongest and most consistent associations in our study were found in this BP recovery phase. Both 5CST- and HGS-defined probable sarcopenia were negatively associated with SBP and DBP recovery in the 10–20 second phase, with a high degree of statistical significance. This means that those with probable sarcopenia had a slower rate of BP recovery. The strength of the effect for HGS was more than twice that of 5CST. Furthermore, those with HGS-defined probable sarcopenia had significantly lower SBP and DBP as 20, 30, and 40 seconds. Lower SBP recovery at 30 seconds has been found previously to be associated with a greater proportion of orthostatic intolerance symptoms (19). The reduced BP recovery in those with HGS-defined probable sarcopenia could hence increase the risk of falls with resultant risk of fracture (33) and subsequent morbidity and mortality (34). The results also fit with a conceptual model of the skeletal muscle pump, which aids BP recovery and maintenance (11).

The majority of existing studies that investigated HGS and OH (8,13,15,35,36) used oscillometric BP, and therefore they were unable to consider the BP recovery phase. Romero-Ortuno et al. (9) assessed the relationship between frailty and OH with continuous BP and did not find a difference in HGS in those with delayed BP recovery; however, they used a morphological clustering approach rather than a strict OH definition and had a smaller sample (*n* = 442). In terms of 5CST and BP recovery, Mol et al. (37) found, using continuous BP measurement, that slower DBP recovery in the 15–30 and 30–60 second periods poststand, but not SBP recovery, was associated with longer 5CST time in older adults after adjustment for relevant confounders.

In the 20–30 and 30–40 second phases, those with HGS-defined probable sarcopenia had a small but significant positive association with SBP and DBP, suggesting faster BP recovery during these phases. However, given that those participants had, on average, entered those time periods with a lower BP, it is physiologically plausible that their rate of recovery would be greater during these phases.

In the steady state period, postrecovery (40–180 seconds), probable sarcopenia had little (5CST) or no (HGS) association with SBP or DBP. Studies looking at classically measured (oscillometric) OH at 3 minutes have found it to be associated with weak HGS (35,36) and confirmed sarcopenia (15), they however, adjusted for limited confounders, and recruited in outpatient (15,36) or hospital inpatient (35) setting, where the demographics and prevalence of OH, sarcopenia and severe sarcopenia may be different. Soysal et al. (13) did not find a significant relationship between OH at 3 minutes and either probable or confirmed sarcopenia. As regards those studies using beat-to-beat BP, Mol et al. (37) did not find an independent association between 5CST time and SBP or DBP from 60 to 180 seconds. Meanwhile, de Bruïne et al. (38) found that participants with the worst performance on the 5CST had significantly higher odds of OH between 15 and 180 seconds compared to those with the best or intermediate performance. The discrepant findings in the published literature may have much to do with the heterogeneity in the methods used and the definitions employed.

The follow up at wave 5 highlights the negative outcomes of probable sarcopenia. Increased rates of recurrent falls were shown in both the HGS- and 5CST-defined probable sarcopenia groups. Some of those falls may be mediated via the OH pathway; others may be directly associated with probable sarcopenia. Both groups also had higher rates of fear of falling with activity limitation, a significant burden on the quality of life in older people (39). HGS- but not 5CST-defined probable sarcopenia had higher rates of hospital admission and mortality, suggesting that HGS might more precisely identify a cohort at risk of adverse outcomes.

Our results are important as they highlight that probable sarcopenia is a potentially modifiable risk factor for OH. They add to the importance of assessing probable sarcopenia; in addition to its well-known direct risk of falls and adverse outcomes (40), a second pathway is emerging, namely the effects of sarcopenia on the skeletal muscle pump with attendant risk of delayed BP recovery from standing and overt OH, adding further to falls risk. The recently published World Falls Guidelines (41) advise consideration of muscle strength assessment with 5CST or HGS as part of a multifactorial falls assessment but do not include it as a graded recommendation in their guidelines from the working groups, nor do they recommend the diagnosis or treatment of probable sarcopenia as routine in their falls work-up. Our results suggest that given its direct risk of falls and the OH-mediated falls pathway, research is needed on the role of identifying probable sarcopenia and sarcopenia in falls risk assessment clinics, and subsequently on the efficacy of interventions to ameliorate sarcopenia.

It must be stated, however, that the size of the effects reported have been modest. This is in keeping with the relatively small contribution of the skeletal muscle pump (vis-à-vis the main cardiac pump) to BP regulation on standing. Although to the best of our knowledge, there exist no published quantitative data on the exact numerical contribution of the skeletal muscle pump to orthostatic BP, a study looking at muscle pumping maneuvers (tip-toeing), in healthy participants, found an average increase of 17 ± 9 mmHg SBP (42). It is likely that the BP rise from quiet standing is significantly less than this and closer to the nonsignificant 4 ± 7 mmHg found with leg crossing in the study. Indeed, baroreflex-mediated heart rate increases and splanchnic vasoconstriction likely contribute to the bulk of BP recovery and maintenance during quiet standing (11).

There are a number of limitations to this study that must be considered. Most significantly, as no measurement of muscle mass was obtained in the TILDA study, we cannot differentiate between participants with probable and confirmed sarcopenia. Therefore, there is the possibility that weak HGS reflected pathological processes other than sarcopenia, such as rheumatological or neurological causes. However, this is tempered by the fact that in clinical practice, HGS assessment is significantly easier to implement than direct measurement of muscle mass with dual-energy x-ray absorptiometry or bioimpedance analysis, perhaps, therefore, making the results more generalizable. This was also an observational study, and unmeasured confounding remains a possibility, although every effort was taken to minimize this. Diseases and medications were self-reported and, therefore, subject to recall bias. In addition, while beat-to-beat BP measurement is the state of the art in terms of assessment of orthostatic BP response, there do exist limitations and challenges with its employment. Previous research has shown low to moderate intraperson reliability of absolute measures, however not on relative measures of changes from baseline (43) as were used in our study. Furthermore, patterns of hemodynamic response to standing have been found to vary over time (44). Factors such as hydration status, meal timing, and caffeine intake can affect results (45), none of which we were able to control for. Comorbidities and medications can also influence BP response and were controlled for in our study, although not the timing of medications in relation to testing.

In summary, probable sarcopenia had a significant association with delayed BP recovery, with HGS-defined probable sarcopenia having a greater effect than 5CST-defined probable sarcopenia. HGS-defined probable sarcopenia was also an independent predictor of OH30. Future studies should investigate if this relationship is maintained for confirmed sarcopenia with direct measurement of muscle mass.
